# Associations of low-carb diets with glycaemic control and diabetic complications among adult Ghanaians: the RODAM study

**DOI:** 10.1007/s00394-025-03729-3

**Published:** 2025-06-07

**Authors:** Tracy B. Osei, Hibbah Osei-Kwasi, Mary Nicolaou, Erik Beune, Charles Agyemang, Karlijn A. C. Meeks, Silver Bahendeka, Matthias B. Schulze, Kerstin Klipstein-Grobusch, Juliet Addo, Charles F. Hayfron-Benjamin, Ina Danquah

**Affiliations:** 1 Heidelberg Institute of Global Health (HIGH), Faculty of Medicine and University Hospital, Heidelberg University, Im Neuenheimer Feld 130.3, 69120 Heidelberg, Germany; 2 School of Sports, Exercise and Health Sciences, Loughborough University, Loughborough, UK; 3 Department of Public and Occupational Health, Amsterdam UMC, University of Amsterdam, Amsterdam Public Health Research Institute, Amsterdam, The Netherlands; 4 John Hopkins University, Maryland, USA; 5 Center for Research on Genomics and Global Health, National Human Genome Research Institute, National Institutes of Health, Bethesda, MD USA; 6 MKPGMS-Uganda Martyrs University, Kampala, Uganda; 7 Department of Molecular Epidemiology, German Institute of Human Nutrition Potsdam-Rehbruecke, Nuthetal, Germany; 8 Institute of Nutritional Science, University of Potsdam, Nuthetal, Germany; 9 Julius Center for Health Sciences and Primary Care, Julius Global Health, University Medical Center Utrecht, Utrecht University, Utrecht, The Netherlands; 10 Division of Epidemiology and Biostatistics, School of Public Health, Faculty of Health Sciences, University of the Witwatersrand, Johannesburg, South Africa; 11 Department of Epidemiology and Population Health, London School of Hygiene and Tropical Medicine, Keppel Street, London, UK; 12 Department of Physiology, University of Ghana Medical School, Accra, Ghana

**Keywords:** Low carbohydrate diet, Diabetes, Glycaemic control and HbA1C

## Abstract

**Purpose:**

We aimed to determine the associations of low-carb diets with glycated haemoglobin (HbA1c) and diabetes complications among Ghanaian adults, who traditionally rely on carbohydrate-dense diets and experience a high prevalence of type 2 diabetes (T2DM).

**Methods:**

This analysis used baseline data of the multi-centre RODAM (Research on Obesity and Diabetes among African Migrants) Study among Ghanaian adults (*N* = 5,898; 18–96 years) living in Ghana and Europe. Energy (kcal/d) and macronutrient intakes (energy%) were computed from the semi-quantitative Ghana Food Propensity Questionnaire. A low-carb diet score (0–30 points) was calculated as the sum of 0–10 points for 11 strata of carbohydrate, protein and fat intakes, respectively. For the associations with ln-transformed HbA1c, we calculated multiple-adjusted beta coefficients, 95% confidence intervals (CIs), and p-values by linear regressions and stratified by T2DM status. Among participants with T2DM, multiple-adjusted odds ratios (OR), 95% CIs, and p-values were computed by logistic regression for the associations of the low-carb diet score with microvascular and macrovascular complications.

**Results:**

Neither macronutrient intakes nor the low-carb diet score were associated with ln (HbA1c) among individuals with T2DM. Among individuals without T2DM, the corresponding associations were statistically significant with marginal beta coefficients between|0.01| and|0.04|. Regarding diabetes complications, we observed an inverse association of the low-carb diet score with self-reported stroke (adjusted OR: 0.95; 95% CI: 0.91, 0.99).

**Conclusion:**

Using a low-carb diet score, our results from this Ghanaian study population do neither support the hypothesis that low-carb diets improve blood glucose control, nor that low-carb diets are associated with diabetes complications.

**Supplementary Information:**

The online version contains supplementary material available at 10.1007/s00394-025-03729-3.

## Introduction

In sub-Saharan Africa and among African migrants living in Europe, type 2 diabetes mellitus (T2DM) is highly prevalent, ranging between 4 and 15% [1]. This high burden has been attributed to sudden lifestyle changes from rapid economic growth, accelerating urbanization, and drastic environmental changes [1]. Diet quality and food quantity play an important role in the development and progression of T2DM among African populations under nutrition transition [1, 2]. In addition, these population groups exhibit context-specific differences in the management and control of T2DM [3]. In previous studies, we have shown that diabetes complications among non-migrant Ghanaians are mainly due to poor glycaemic control [4]. Also, in urban Ghana, the high prevalence of microvascular complications among patients with T2DM is a major concern [5].

With respect to the management of T2DM, dietary recommendations for carbohydrates have been an area of continual controversy until today [6]. Before the availability of insulin, a low-caloric diet based on restricted carbohydrate intake, but high dietary fat was recommended [7]. This was based on the hypothesis that low-carb diets reduce postprandial glucose responses. Hence, low-carb diets help to improve insulin sensitivity and reduce hyperglycaemia [8, 9]. Indeed, post-prandial hyperglycaemia constitutes a risk factor for the development of various metabolic pathologies and hospital mortality, even in individuals without T2DM [10, 11]. Furthermore, there is also growing evidence, suggesting that low-carb diets may be more effective for weight-loss [9, 12]. On the other hand, low-carb diets may compromise the full-range supply of nutrients, potentially leading to deficiencies of vitamins and minerals, such as vitamin C, calcium, potassium and copper [13]. In addition, there are concerns that low-carb diets may increase the risk of some diabetes complications such as nephropathy, stroke, peripheral artery disease and coronary artery disease, due to higher intakes of dietary fat. Particularly, saturated fat is associated with increased blood levels of low-density lipoprotein (LDL)-cholesterol and vascular dysfunction [9, 14]– important risk factors for the above-mentioned cardio-vascular conditions.

Irrespective of this controversy, the maintenance of glycaemic control constitutes one of the most important approaches for the effective management of T2DM [15]. To achieve this, diet therapy and health promotion strategies are essential to improve and stabilize concentrations of glycated haemoglobin (HbA1c)– the indicator for long-term glucose control [16]. In fact, systematic reviews and meta-analyses show the health-beneficial effects of low-carb diets among individuals with T2DM with European ancestry in North America and Australia, comprising rapid improvements in HbA1c, subsequent reduction in the use of glucose-lowering medication, significant weight-loss, and improved lipid profile [17, 18]. Still, the health effects of low-carb diets among individuals from sub-Saharan Africa remains to be verified.

Notably, among individuals from Ghana, the diets traditionally are based on carbohydrate-dense staple foods such as plantain, cassava and maize [19]. Also, carbohydrate-dense snacks are mostly included in the diet upon urbanization and migration to Europe [20]. Therefore, the recommendation of low-carb diets for the management of T2DM may seriously challenge culinary traditions and dietary habits.

Considering the above-mentioned constellation of (i) a high burden of T2DM and its complications among individuals from Ghana, (ii) the conflicting evidence regarding low-carb diets for T2DM management, and (iii) the reliance on carbohydrate-rich foods in this population, we aimed at establishing the associations of low-carb diets with HbA1c and selected diabetes complications among Ghanaian adults living in five different geographic locations.

## Methods

### Study design and population

An elaborate description of the study design and methods of the Research on Obesity and Diabetes among African Migrants (RODAM) Study has been published elsewhere [21]. In brief, the RODAM study is a multi-centre, cross-sectional study that aims to investigate the interplay between environmental and genetic risk factors and their relative contributions to obesity and T2DM among Ghanaian migrants in Europe and their counterparts living in rural and urban Ghana. Overall, 6,385 participants between the ages of 18 and 96 years were recruited between July 2012 and September 2015. For recruitment, in Ghana, census data of 2010 were used to draw rural and urban participants in the Ashanti Region. In Amsterdam, the Municipal Register was used to randomly select Ghanaian migrants who were then invited by postal mail and home visits. In London and Berlin, Ghanaian organizations, church communities and social unions served as the sampling frame for recruitment. Of those invited to participate, the participation rate was 76% in rural Ghana (15 villages), 74% in urban Ghana (Kumasi and Obuasi), 75% in London, and 68% in Berlin. In Amsterdam, a response to the invitation to participate was received from 67% and, of these, 53% agreed to participate. About 99% of the Ghanaian participants in Europe were first-generation migrants.

#### Ethical approvals

were obtained from the local ethics committees in Ghana (CHRPE/AP/200/12), United Kingdom (#6208), The Netherlands (#W12_062), and Germany (EA1/307/12). All participants gave informed written consent, before their enrolment in the study.

### Data collection procedures

As a central feature of the RODAM study, at all study sites, the same standard operating procedures (SOPs) were used for data collection. We performed questionnaire-based interviews to collect socio-demographic, lifestyle, and medical history data. Interviewers speaking the Ghanaian local languages were thoroughly trained, and their performance was monitored during the initial interviews. Feedback was provided, and changes were initiated if needed [21]. Physical examinations were performed, including anthropometric measurements and blood pressure and pulse measurements. Fasting venous blood samples and morning urine samples were collected for biochemical analysis.

### Assessment of biochemical markers

Fasting venous blood samples were collected by trained research assistants in all sites. All the blood samples were processed and aliquoted immediately (within 1 h to maximum 3 h of the vena puncture) after collection according to standard operation procedures, and then temporarily stored at the local research location at − 20 °C. The separated samples were then transported to the local research centres’ laboratories, where they were checked, registered and stored at − 80 °C. To avoid intra-laboratory variability, the stored blood samples from the local research centres were transported to Berlin for biochemical analyses.

Plasma concentrations of fasting blood glucose (FPG) were measured using an enzymatic method (hexokinase). High-performance liquid chromatography (TOSOH G8 HPLC analyzer) was used to measure HbA1c, reported as mmol/mol. HbA1c was used as a single biomarker for glycaemic control. T2DM was defined according to the guidelines of the World Health Organization (WHO) as fasting plasma glucose ≥ 7.0 mmol/L or HbA1c ≥ 48 mmol/mol (≥ 6.5%) or documented use of glucose-lowering medication or self-reported diabetes. Immuno-turbidimetric assays were used to measure the serum lipid profile (total cholesterol, triglycerides, high-density lipoprotein (HDL)-cholesterol, low-density lipoprotein (LDL)-cholesterol).

Serum creatinine concentration was determined by a kinetic colorimetric spectrophotometric isotope dilution mass spectrometry calibration method (Roche Diagnostics). The estimated glomerular filtration rate (eGFR) was calculated using the 2009 CKD-EPI (CKD Epidemiology Collaboration) creatinine equation, and the kidney disease severity was measuring using the 2012 KDIGO guidelines [22]. Direct analyses of urinary albumin and creatinine concentration were performed on an early morning urine sample. Urinary albumin concentration was measured by an immunochemical turbidimetric method (Roche Diagnostics). Urinary creatinine concentration (in µmol/L) was measured by a kinetic spectrophotometric method (Roche Diagnostics). Urinary albumin-creatinine ratio (ACR) was calculated and expressed in mg/mmol.

### Assessment of diabetes complications

We included nephropathy as a microvascular complication. This was defined based on urinary albumin and eGFR [22]. Albuminuria and eGFR were classified using the 2012 kidney disease: Improving Global Outcomes (KDIGO) guidelines: A1 indicates normal to mildly elevated levels (< 3 mg/mmol), A2 indicates substantially elevated levels (3–30 mg/mmol), and A3 indicates significantly elevated levels (> 30 mg/mmol). Low eGFR was defined as eGFR < 60 mL/min/1.73 m^2^ (severely increased) [23]. Because of the small number of cases in the A3 category, albuminuria was defined using A2 and A3 categories, (ACR ≥ 3 mg/mmol). Accordingly, nephropathy was defined as reduced eGFR or the presence of albuminuria or both. Macrovascular complications comprised peripheral artery disease (PAD), coronary artery disease (CAD), and self-reported stroke. Systolic and diastolic blood pressure were measured three times, after 5-min resting time and while being seated. We used appropriately sized cuffs and a certified semi-automated device (MicrolifeWatch BP home, Widnau, Switzerland). For the analysis, we used the mean of the last two measurements.

PAD was assessed by the ankle brachial pressure index (ABI) as the ratio of the resting systolic blood pressure at the ankle to the resting systolic brachial pressure at the arm. This was obtained from the blood pressure on both the right and left side of the legs and arms using the Microlife Watch BP Office. PAD was defined as ABI ≤ 0.90 [24]. CAD was assessed using the WHO Rose angina questionnaire. Angina was defined as a positive reply to the questions ‘Have you ever had any pain or discomfort in your chest?’ and ‘Do you get this pain or discomfort when you walk uphill or hurry?’ Stroke was defined by a positive reply to the question ‘Have you ever had a stroke?’.

### Dietary assessment

Detailed explanation of the methods of dietary assessment have previously been published [19]. Food intake was assessed with a standardised semi-quantitative Ghana-specific food propensity questionnaire (Ghana-FPQ) that queried for the usual intake of 134 food groups during the past 12 months. Standardised description of portion sizes was facilitated using common Ghanaian household utensils. The Ghana-FPQ was calibrated using 24 h dietary recalls, which were collected from a random sub-sample of the study population [19]. Here, calibration refers to the identification of representative foods in the food groups, site-specific portion sizes, and meal recipes. Ghana-FPQ data were translated into energy intakes (kcal/day) and the intakes of carbohydrates, fats, protein, and fiber (g/day) by applying the West African Food Composition Table (2012) and the German Nutrient Database (BLS, 2010).

### Assessment of covariates

Physical examination was conducted with validated devices according to the same SOPs across all study sites. All anthropometric measurements were taken twice by the same assessor, and the means of the two measurements were used for analyses. Body weight (kg) was taken in light clothing and without shoes using a weighing scale, height (cm) by a stadiometer, and waist circumference (cm) using a measuring tape (all devices SECA, Germany). Body Mass Index (BMI) was calculated as weight over height squared (kg/m^2^).

Data on socio-demographics, medical history, and lifestyle factors were obtained through a structured self-administered questionnaire, or a face-to-face interview by well-trained study personnel. Educational status of participants was categorised as never/elementary, lower, intermediate, and higher/tertiary level. The WHO Stepwise approach to chronic disease risk factor Surveillance (STEPS) questionnaire [25] was used to derive physical activity in metabolic equivalent h/week, which included physical activity at work, while commuting, and in leisure time. Answers were subsequently classified based on the guidelines of the international physical activity questionnaires for adults (IPAQ) group into three levels of total physical activity (low, moderate, high) [26]. Smoking status was categorised as current, former, or non-smokers.

### Data analysis

#### Missing data handling

Data processing and analysis was done using the Statistical Analysis Software SAS (version 9.4) [27]. Out of 6,385 participants, 487 neither attended the physical examinations nor provided blood samples. As a result, 5,898 participants were included in the analysis. Of these, 662 had T2DM and 5,298 did not. In total, 2,537 participants had missing data on at least one of the variables of interest (T2DM: 284; no-diabetes: 2,253). We applied multiple imputation (*n* = 10; discriminant fully conditional specification (FCS) method) for missing data of the variables of interest, in order to increase the statistical power and to reduce potential selection bias [28]. Considering that the data contain many patterns of missing values, and both are categorical and continuous, we applied the FCS method [29]. Imputation was done for both exposure and outcome variables. The flow chart for missing and imputed variables by T2DM status is presented in Supplementary Figure S1. The final analytical sample size comprised 3,361 participants (T2DM: 378; non-diabetes: 2,983). The characteristics of the participants in the complete-case dataset are shown in Table S1.

#### Operationalization of low-carb diets

We used the approach by Halton et al. (2006) to operationalize low-carb diets [30]. Participants were divided into deciles based on the percentages of energy consumption from fat, protein, and carbohydrates (Supplementary Table S2). We used the nutrient deciles in the non-diabetes group to minimise the risk of reverse causation. This refers to the possibility that individuals with long-standing T2DM might have changed their diet. For fat and protein, participants in the highest decile received a score of 10 points and in the lowest stratum, they received 0 points. Conversely, for carbohydrates, the allocation of score points were the opposite. An overall low-carb diet score ranging from 0 to 30 was created by summing the points for the three macronutrients. To depict dietary composition and minimize bias caused by underreporting food consumption, we used the proportion of energy consumed rather than the absolute intake. A higher score indicated a higher proportion of energy from total fat and total protein and a lower proportion of energy from carbohydrates.

#### Association analyses

In order to comment on possible selection bias, all association analyses were performed in the imputed dataset (10 datasets) and the complete-case dataset, which contained those participants who had complete data for all relevant variables. In the main manuscript, we present the findings from the imputed dataset; the results from the complete-case dataset are shown in the supplementary material. We stratified all analyses by T2DM status, because of the presumably different impacts of low-carb diets among individuals with or without T2DM, particularly among those who are on glucose-lowering medication. Multiple-adjusted linear regression models were calculated for natural log-transformed HbA1c as a continuous outcome, and using energy intake, macronutrients consumption (total carbohydrates, protein, total fat), and the low-carb diet score as continuous exposure variables. We adjusted for sociodemographic and lifestyle factors in model 1 (age, sex, and study site) and model 2 (model 1 + educational level, smoking status, physical activity, and fibre diet) respectively due to their influence on T2DM and dietary intake. We also added fibre intake to model 2 because of its impact on the quality of carbohydrate intake. The PROC MIANALYZE procedure in SAS was used to calculate ß-coefficients, their 95% confidence intervals (CIs), and p-values per one standard deviation (SD) increase of the exposure variables. Finally, logistic regression models were calculated for the associations of the low-carb diet score with diabetes complications among participants with T2DM, using the same set of potential confounders (Models 1 and 2). Residuals were inspected for normal distribution to ensure that assumptions for linear regression analyses were met.

Additionally, we conducted several sensitivity analyses to verify the robustness of our findings. First, we assessed the potential of reverse causation by excluding 222 individuals with self-reported T2DM (*n* = 440). In this dataset, we calculated the associations of the low-carb diet score, energy intake, and macronutrient intakes with ln(HbA1c) and with diabetes complications. Second, we tested interactions with study sites for the associations between dietary factors and HbA1c using multiplicative interaction terms. The proportions of diabetes complications were too low to conduct a site-stratified analysis for these outcomes.

## Results

### Characteristics of the study population

Socio-demographic, anthropometric, lifestyle, and clinical characteristics of the RODAM study population, by sex and by study site, are presented in Table 1. The mean age (46.1 ± 11.9 years) was similar across study sites, with the lowest mean age in Berlin and the highest mean age in rural Ghana. There was a preponderance of women (62.2%). Men generally had a higher level of education and physical activity and were more likely to drink alcohol and smoke as compared to women. Across sites, the proportion of participants with advanced education was highest in London. Mean BMI (25.2 ± 4.4 kg/m^2^ vs. 28.3 ± 5.7 kg/m^2^) and waist circumference (87.2 ± 12.1 cm vs. 91.9 ± 12.8 cm) were lower in males than in women. Participants in London showed the lowest level of physical activity but the highest BMI and waist circumference. In men, HbA1c (median: 37.1; IQR: 12.2–41.5 mmol/mol) was higher than in women (median: 36.8; IQR: 11.5-152.5 mmol/mol), and this was also seen for the crude prevalence of T2DM (men: 13.1% vs. women: 10.8%). Across the study sites, the highest proportions of T2DM were seen in Berlin (14.5%), followed by Amsterdam (13.8%), London (12.7%), urban Ghana (10.5%), and rural Ghana (5.2%).

**Table 1:** Characteristics and biomarkers of the RODAM study population by sex and site

Characteristics	Total	Men	Women	Rural Ghana	Urban Ghana	Amsterdam	Berlin	London
	(*n* = 5898)	(*n* = 2229)	(*n* = 3669)	(*n* = 1111)	(*n* = 1455)	(*n* = 1633)	(*n* = 578)	(*n* = 1121)
Sex (female, %)	3669 (62.2)	0 (0.0)	3669 (100)	693 (62.4)	1046 (71.9)	972 (59.5)	260 (45.0)	661 (59.0)
Age (years)	46.1 ± 11.9	46.8 ± 12.2	45.7 ± 11.7	48.4 ± 14.4	45.3 ± 11.5	45.3 ± 10.7	44.4 ± 11.5	47.0 ± 11.2
**Education (%)**								
Never or elementary	2022 (34.3)	21 (21.0)	1553 (42.4)	650 (58.5)	633 (43.5)	557 (34.1)	51 (8.8)	131 (11.7)
Low	2148 (36.5)	884 (39.8)	1263 (34.4)	337 (30.3)	567 (39.0)	589 (36.1)	290 (50.2)	365 (32.5)
Intermediate	1082 (18.3)	503 (22.5)	579 (15.8)	83 (7.5)	182 (12.5)	381 (23.4)	160 (27.6)	276 (24.6)
Higher vocational	646 (11.0)	373 (16.8)	274 (7.5)	42 (3.7)	73 (5.0)	106 (6.5)	77 (13.3)	349 (31.2)
Body mass index (kg/m^2)^	27.1 ± 5.5	25.2 ± 4.4	28.3 ± 5.7	22.5 ± 4.2	26.9 ± 5.3	28.7 ± 5.0	27.5 ± 4.8	29.5 ± 4.9
Waist circumference (cm)	90.3 ± 12.7	87.2 ± 12.1	91.9 ± 12.8	81.1 ± 10.7	89.3 ± 11.9	93.9 ± 12.3	91.8 ± 11.5	95.0 ± 11.8
Type 2 diabetes (positive)	662 (11.2)	292 (13.1)	370 (10.8)	58 (5.2)	152 (10.5)	225 (13.8)	84 (14.5)	142 (12.7)
HbA1c (mmol/mol)	36.8 (11.5, 163)	37.1 (12.2, 41.5)	36.8 (11.5, 152.5)	31.5 (27.3, 35.7)	35.7 (31.6, 39.9)	38.9 (34.7, 42.7)	38.0 (34.4, 41.9)	39.9 (35.6, 43.1)
Smoking (current/ former, %)	595 (10.1)	450 (20.2)	145 (4.0)	104 (9.3)	103 (7.1)	208 (12.8)	115 (19.9)	66 (6.0)
Physical activity (MET-min/day)	60 (11, 161)	84 (16, 189)	51 (8, 144)	84 (28, 163)	60 (6, 156)	72 (16, 200)	64 (11, 195)	25 (0, 108)
Triglycerides (mmol/L)	0.87 (0.66, 1.19)	0.92 (0.70, 1.23)	0.84 (0.64, 1.16)	0.98 (0.74, 1.31)	1.01 (0.75, 1.35)	0.77 (0.59, 1.04)	0.85 (0.64, 1.13)	0.78 (0.61, 1.05)
LDL-cholesterol (mmol /L)	3.2 ± 1.0	3.1 ± 1.0	3.2 ± 1.0	2.8 ± 1.0	3.4 ± 1.0	3.2 ± 0.9	3.2 ± 1.1	3.2 ± 0.9
HDL-cholesterol (mmol/L)	1.3 ± 0.4	1.3 ± 0.4	1.4 ± 0.4	1.2 ± 0.4	1.3 ± 0.3	1.4 ± 0.3	1.5 ± 0.4	1.4 ± 0.3
**Albuminuria category**								
Normal: <3 mg/mmol	5376 1.2)	2061 (92.5)	3314 (90.3)	1014 (91.3)	1294 (88.9)	1521 (93.1)	516 (89.3)	1031 (91.8)
Moderate: 3–30 mg/mmol	460 (7.8)	143 (6.4)	317 (8.4)	86 (7.7)	139 (9.6)	99 (6.06)	57 (9.9)	79 (7.1)
Severe: ˃30 mg/mmol	62 (1.5)	24 (1.8)	38 (1.0)	11 (1.0)	22 (1.5)	13 (0.8)	5 (0.9)	11 (1.0)
CKD-EPI eGFR (mL/min/1.73 m^2^)	95.2 ± 20.3	94.4 ± 19.8	95.6 ± 20.6	96.7 ± 22.7	93.2 ± 19.9	96.6 ± 20.2	98.9 ± 19.5	92.1 ± 17.9

Continuous variables are expressed as means ± standard deviations (SDs) or medians (interquartile range, IQR). Categorical variables are expressed as numbers (percentages). CKD-eGFR: chronic kidney diseases according to estimated glomerular filtration rate; HDL: high-density lipoprotein; LDL: low-density lipoprotein; MET: metabolic equivalent of task; Type 2 diabetes was defined as fasting plasma glucose ≥ 7.0 mmol/L or HbA1c ≥ 48 mmol/mol (≥ 6.5%) or documented use of glucose-lowering medication or self-reported diabetes mellitus; HbA1c: glycated haemoglobin

With respect to dietary characteristics (Table 2), carbohydrates, total fat, and protein contributed 53.2 ± 9.3 energy%, 32.3 ± 8.3 energy%, and 13.5 ± 2.7 energy%, respectively. These contributions were similar between men and women, although males had higher mean energy intake compared to females. Across sites, carbohydrate intakes were highest in urban Ghana, followed by rural Ghana, Amsterdam, London, and Berlin. Conversely, the relative contributions of dietary fat to energy intake were higher in Europe than in Ghana. This was also true for protein intakes. These distributions were reflected in the means of the low-carb diet score across study sites with the highest mean score in London and the lowest in rural Ghana. In addition, these substitutional relationships between the low-carb diet score and macronutrient contributions to energy intake are reflected in the partial Pearson correlations in Table S3. The characteristics were similar between the imputed dataset and the complete-case dataset (Table S1).

**Table 2:** Dietary characteristics of RODAM study population by sex and site

Characteristics	Total	Men	Women	Rural Ghana	Urban Ghana	Amsterdam	Berlin	London
N	5,898	2,229	3,669	1,111	1,455	1,633	578	1,121
Carbohydrate intake (energy%)	53.2 ± 9.3	52.4 ± 9.5	53.6 ± 9.0	54.6 ± 8.2	57.5 ± 8.8	51.6 ± 8.2	49.5 ± 10.6	51.4 ± 9.1
Fat intake (energy%)	32.3 ± 8.3	32.2 ± 8.6	32.3 ± 8.1	30.5 ± 7.7	31.5 ± 7.3	32.6 ± 8.2	33.9 ± 10.3	33.6 ± 8.6
Protein intake (energy%)	13.5 ± 2.7	13.4 ± 2.7	13.5 ± 2.7	11.3 ± 2.3	13.5 ± 2.5	14.4 ± 2.5	13.6 ± 2.6	14.0 ± 2.5
Total energy (kcal/day)	2714.3 ± 116.5	2857.5 ± 1240.9	2627.3 ± 1106.5	2948.4 ± 1414.7	2335.0 ± 747.6	2612.9 ± 1067.1	3235.9 ± 1420.8	2853.3 ± 1144.7
Low-carb diet score	15.2 ± 7.8	15.3 ± 7.8	15.1 ± 7.8	10.4 ± 7.7	14.7 ± 7.5	16.9 ± 6.9	16.8 ± 7.8	17.2 ± 7.4
Alcohol intake (g/day)	0.1 (0, 2.0)	0.8 (0, 5.5)	0.1 (0, 1.2)	0.1 (0, 1.2)	0.1 (0, 0.6)	1.3 (0, 5.2)	1.8 (0, 10.9)	0.1 (0, 1.6)

Continuous variables are expressed as means ± standard deviations (SDs) or median (interquartile range, IQR). Categorical variables are expressed as percentages (%)

### Diabetes complications

**Fig. 1:**
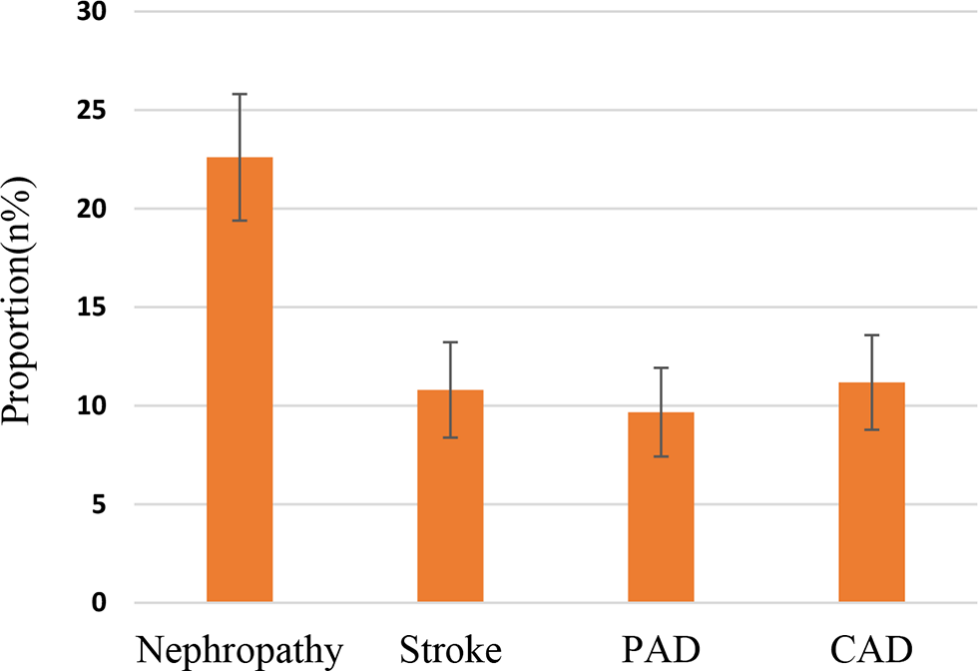
Crude proportions (%) of diabetes complications among participants with type 2 diabetes (*n* = 662). Error bars indicate 95% confidence intervals (CIs). Stroke was self-reported. PAD: Peripheral Artery Disease; CAD: Coronary Artery Disease

Figure 1 shows the crude proportions of microvascular and macrovascular complications among 662 participants with T2DM. Overall, the most frequent complication was nephropathy 22.6% (95% CI: 0.19, 0.26), followed by CAD 11.2% (0.09, 0.14), self-reported stroke 10.8% (0.08, 0.13), and PAD 9.7% (0.07, 0.12). These proportions were similar in the complete-case dataset (Figure S2).

### Associations with HbA1c

For individuals with and without T2DM, we present the associations of the low-carb diet score, total energy intake, and macronutrients with ln(HbA1c) in Table 3. While there were statistically significant associations in the subgroup without T2DM, the corresponding beta-coefficients for HbA1c per 1 SD increase in the exposure variables were small, ranging from|0.01| to|0.04|. Specifically, the associations were positive for the low-carb diet score (beta: 0.01; 95% CI: 0.00, 0.02), protein intake (beta: 0.01; 95% CI: 0.01, 0.02), and total fat intake (beta: 0.01; 95% CI: 0.00, 0.01). However, for carbohydrate intake, there was an inverse association with HbA1c (beta: -0.01; 95% CI: -0.01, 0.00).

**Table 3:** Linear associations of low-carb diet score, total energy intake and the intakes of macronutrients with Ln (HbA1c) among 5,898 Ghanaian adults, by diabetes status

ln (HbA1c)	SD	*Crude model*		*Model 1*		*Model 2*	
		Std ß (95% CI)	*p*-value	Std ß (95% CI)	*p*-value	Std ß (95% CI)	*p*-value
**Diabetes (*n*** **= 662)**							
Low-carb diet score (per 1 SD)	7.6	-0.002 (-0.03, 0.03)	0.881	0.02 (-0.01, 0.05)	0.311	0.02 (-0.02, 0.05)	0.370
Energy intake (per 1 SD) (kcal/day)	1090	-0.02 (-0.05,0.10)	0.204	-0.01 (-0.04,0.02)	0.391	0.05 (-0.02,0.12)	0.157
Carbohydrate (per 1 SD) (energy%)	9.7	0.01 (-0.02, 0.04)	0.533	-0.01 (-0.04, 0.02)	0.619	-0.01 (-0.05, 0.02)	0.494
Protein (per 1 SD) (energy%)	2.7	0.01 (-0.02, 0.03)	0.648	0.02 (-0.01, 0.05)	0.116	0.02 (-0.02, 0.06)	0.385
Total fat (per 1 SD) (energy%)	8.8	-0.01 (-0.03, 0.02)	0.653	0.002 (-0.03, 0.03)	0.906	0.01 (-0.03, 0.05)	0.569
**No diabetes (*n*** = **5,236)**							
Low-carb diet score (per 1 SD)	7.8	0.03 (0.02, 0.04)	< 0.0001	0.02 (0.01, 0.02)	< 0.0001	0.01 (0.00, 0.02)	0.007
Energy intake (per 1SD) (kcal/day)	1178	-0.01 (-0.01,0.001)	0.073	-0.002 (-0.01,0.004)	0.505	0.01 (0.00,0.03)	0.062
Carbohydrate (per 1 SD) (energy%)	9.2	-0.03 (-0.3, -0.02)	< 0.0001	-0.01 (-0.02, -0.01)	< 0.0001	-0.01 (-0.01, 0.00)	0.037
Protein (per 1 SD) (energy%)	2.7	0.04 (0.03, 0.04)	< 0.0001	0.02 (0.01, 0.02)	< 0.0001	0.01 (0.01, 0.02)	0.0003
Total fat (per 1 SD) (energy%)	8.2	0.02 (0.01, 0.02)	< 0.0001	0.01 (0.01, 0.02)	0.0004	0.01 (0.00, 0.01)	0.046

Standardized (Std) beta-coefficients (β), 95% confidence intervals (CIs), and p-values were calculated by linear regression. SD: standard deviation

Model 1: adjusted for age (years), sex, study site (5 categories)

Model 2: Model 1 + education (4 categories), smoking (yes/no), physical activity (MET-h/week), and dietary fibre (g/d)

In fact, the point estimates were similar in the subgroup with T2DM but were not statistically significant. This lack of association was seen for all exposures of interest, including the low-carb diet score. The same results were produced from the complete-case dataset (Table S4). In the sensitivity analysis, we excluded 222 participants with self-reported diabetes to rule-out reverse causation. The lack of associations of the low-carb diet score, total energy intake, and the intakes of macronutrients with ln(HbA1c) remained among 440 participants with screen-detected T2DM (Table S5).

Additionally, we tested interactions with study site for the associations of the low-carb diet score, energy intake, and macronutrient intakes with ln(HbA1c) (Table S6). Among individuals without diabetes mellitus (Ghana: 174; Europe: 204), the tests for interaction were significant for the low-carb diet score (*p* = 0.011), carbohydrate intake (*p* = 0.031), and protein intake (*p* = 0.002). In Europe, there were null associations; in Ghana, the low-carb diet score was positively associated with ln(HbA1c) (std. beta: 0.06; 95% CI: -0.003, 0.13) and conversely, carbohydrate intake was inversely associated with ln(HbA1c) (std. beta: -0.07; 95% CI: -0.14, -0.001). Among individuals with T2DM (Ghana: 1,788; Europe: 1,195), we also found significant interactions for the low-carb diet score (*p* < 0.0001) and macronutrient intakes (Table S6). The same inverse association was seen for carbohydrate intake in Ghana (std. beta: -0.01; 95% CI: 0.005, 0.02) but not in Europe. Correspondingly, total fat intake showed the opposite associations with ln(HbA1c) (Table S6).

### Associations with diabetes complications

Table 4 shows the associations between the low-carb diet score and diabetes complications among individuals with T2DM (*n* = 662). In the final model, we observed a 5% reduced chance of self-reported stroke for each SD increase in the low-carb diet score (95% CI: 0.91, 0.99; *p* = 0.014). In the complete-case dataset, the corresponding value was 7% (95% CI: 0.87, 0.98; *p* = 0.004) (Table S7). There were no associations of the low-carb diet score with PAD, CAD, and nephropathy– neither in the imputed dataset nor in the complete-case dataset (Table 4, Table S7). Again, we found that for every SD increase in the low-carb diet score (91% CI: 0.87, 0.96; *p* = 0.014), the likelihood of self-reported stroke decreased by 5% in the dataset that did not include self-reported diabetes (Table S8).

**Table 4:** Multiple-adjusted associations of the low-carb diet score with diabetes complications among 662 participants with diabetes

Outcome	Cases/ non-cases	Crude model				Model 1				Model 2			
		OR	95% CI		*p*-value	OR	95% CI		*p*-value	OR	95% CI		*p*-value
Peripheral artery disease	64/598	0.99	(0.87,	1.13)	0.902	0.99	(0.87,	1.12)	0.901	0.99	(0.99,	1.14)	0.925
Coronary artery disease	74/588	1.01	(0.96,	1.06)	0.730	1.01	(0.96,	1.07)	0.618	1.04	(0.98,	1.10)	0.209
Self-reported stroke	68/594	0.91	(0.88,	0.95)	< 0.0001	0.95	(0.92,	0.99)	0.013	0.95	(0.91,	0.99)	0.014
Nephropathy	157/505	1.08	(0.98,	1.20)	0.114	1.09	(0.98,	1.21)	0.122	1.09	(0.98,	1.21)	0.126

Odds ratios (ORs), their 95% confidence intervals (CIs), and p-values were calculated by logistic regression

Model 1: adjusted for age (years), sex, study site (categorical)

Model 2: Model 1 + education (4 categories), energy intake (kcal/d), smoking (yes/no), physical activity (MET-h/week), and dietary fibre (g/d)

## Discussion

The present study among Ghanaian adults determined the associations of low-carb diets with glycaemic control and diabetes complications. We found no significant associations between the low-carb diet score and HbA1c among participants with T2DM. Among participants without T2DM, the low-carb diet score showed marginal, positive, and statistically significant associations with HbA1c. For diabetes complications, we saw a 5%-reduced chance of self-reported stroke for each SD increase in the low-carb diet score, but no associations with other diabetes complications.

### Associations of low-carb diet score with glycaemic control

Dietary guidelines for clinical practice acknowledge the important role of dietary macronutrients for maintaining the glucose-insulin balance [6, 7]. In our study, macronutrient intakes and the low-carb diet score were barely associated with HbA1c among participants with T2DM. Although, our findings may be influenced by the use of glucose-lowering medication in some of the participants with T2DM, earlier studies have shown that low-carb diets are beneficial for glycaemic control [6, 7]. However, a recent systematic literature review and meta-analysis of 102 controlled feeding trials demonstrated a lack of effect by fats and carbohydrates on blood glucose control and the onset of T2DM [31]. It can be argued that the principal mechanism by which low-carb diets improve glycaemic control lies not only in the reduction of carbohydrates but rather in the quality of the carbohydrates. Indeed, low-carb diets that feature complex carbohydrates, such as whole-grain foods, have a lower glycaemic index and glycaemic load and thus, are beneficial to blood glucose control compared to high-carb diets [30]. In fact, better carbohydrate quality and higher proportions of saturated fats in Ghana [19, 20] could partially explain the direct association between the low-carb diet score and HbA1c in Ghana but not in Europe. Some studies have indicated that low-carb diets may not be characterized by whole-grains and lower glycaemic load, because they enforce food choice restrictions [32]. For instance, when following low-carb diets, fruits and vegetables are typically consumed in low quantities, which lowers the overall intake of dietary fibre, vitamins and minerals [32]. Again, another prior meta-analyses that reviewed the benefit of low-carb diet in persons with T2DM reported that although, low-carb diets may have larger short-term gains in glycaemic control and weight loss, the long-term efficacy and safety on individuals with T2DM is inconclusive [33]. It is speculated that insulin sensitivity may be negatively affected in the long-term as low-carb diets may stimulate high plasma circulation of free fatty acids. This is associated with insulin resistant states in humans [32]. Indeed, in the UK National Diet and Nutrition Survey (2008–2016) with 3,234 individuals, participants without T2DM, reductions in carbohydrates and increase in total fat intake have been associated with increased HbA1c [8].

### Associations of low-carb diet score with diabetes complications

Although, it has been noted that low-carb diets may exert beneficial effects on the cardiovascular risk factor profile [34], including blood triglycerides and cholesterol, the observed risks of CVD through low-carb diets remain inconsistent among the general public. In a prospective study with 42,237 Swedish women, a low-carb diet was associated with high risk of CVD [35], and this was also seen for the risk of stroke in a large Asian population [36]. In contrast, in the Nurses’ Health study, a low-carb diet was not associated with CVD risk [30], which was explained mainly by the health-beneficial effects of reduced body weight [37]. Possible reasons for the inconsistent association between low-carb diets and the risk of diabetes complications in our study may lie in the quality of macronutrients. Epidemiological studies have established that the complexity of carbohydrates and the quality of fats have different impacts on cardiovascular risk [30]. Refined carbohydrates are associated with increased risk for both, ischemic and haemorrhagic stroke [38], while whole-grains, fruits and vegetables have shown protective features against ischemic stroke [39]. Another study showed an inverse association of low-carb diets, based on plant protein and plant fat, with CVD mortality [40].

It has been suggested that low-carb diets may provide metabolic advantages such as favouring weight loss, and increasing the reliance on fatty acid oxidation for energy production, thereby, favouring fat-loss and improving HDL-cholesterol and triglyceride concentrations [41, 42]. However, these advantages remain contentious as some studies have reported increases in more atherogenic lipids (LDL-cholesterol, non-HDL-cholesterol) in individuals with and without T2DM [41]. The explanation of this paradox may include increased absorption of dietary fat or altered lipid metabolism [43]. Exposure to a hyperlipidaemic milieu as a result of high lipolysis and reduced lipogenesis results in the ingestion and processing of lipids, predominantly known as modifiable LDL, by macrophages [44, 45]. Atherosclerosis may develop when these macrophages accumulate to form plaques in the sub-endothelium of the arteries [44], and can ultimately lead to stroke.

Although many studies explicitly refer to low-carb diets as the better nutritional intervention for glycaemic control, this dietary regimen maybe accompanied with elevated fat and protein intake. Hence, any effects observed should consider the parallel change in other macronutrients and any conclusions should be viewed in that light [46].

Therefore, a practical advice for following low-carb diets should be geared towards the reduction of refined carbohydrates but the inclusion of unsaturated fats, plant proteins and vegetables [47].

### Strengths and limitations

As a strength of our analysis, the low-carb diet score allows a practical approach to operationalize a dietary pattern, which reflects the complementarity of other macronutrient intakes rather than focusing on single nutrients, which are never consumed in isolation. Still, we acknowledge that the Ghana-FPQ may not measure absolute dietary intake. Yet, it is useful to rank participants according to their intakes. Therefore, the low-carb diet score was not constructed by using absolute cut-offs as suggested by Halton et al. [30]. Rather, the score was derived based on the distributions of macronutrient intake among the non-T2DM group to account for the imprecision of the assessment tool and to rule-out potential reverse causation among individuals with long-standing T2DM. At the same time, the low-carb diet score only employs the contribution of macronutrients to energy intake. We cannot use it to operationalize the quality of macronutrients, which may be important for both, glucose control and the development of diabetes complications. Regarding the study population, we stratified the analysis by T2DM status using a dichotomous variable. We acknowledge that T2DM is a transient disease that comprises various stages of pre-diabetes. Here, we decided to concentrate on the two clinically relevant sub-groups in order to derive dietary recommendations that can be communicated to healthy individuals and patients with T2DM. Additionally, the cross-sectional nature of the study may give rise to reverse causation and recall bias, particularly among those individuals with self-reported T2DM (*n* = 222). They might have experienced dietary advise, giving rise to biased responses to the diet-related questions. Therefore, we have excluded these subjects in a sensitivity analysis, which produced similar findings. Of note, stroke was only self-reported, which could have led to misclassification and the findings should be interpreted with caution. Also, statistical power due to small numbers of stroke cases might have been an issue. Coronary arteriography was not performed in the evaluation of CAD due to infeasibility. However, the WHO Rose Angina Questionnaire has been proven to be a useful predictor for CAD in many epidemiological studies [48]. HbA1c as a measure of glycaemic control is associated with many caveats: level of haemoglobin, episodes of malaria infection, glucose-lowering drugs, and anti-retroviral therapy. We are confident that multiple imputation has provided us with increased statistical power. There was no indication of selection bias, as the general and dietary characteristics were fairly similar between the imputed dataset and the complete-case dataset.

## Conclusions

This study among Ghanaian adults adds to the growing body of literature about the associations between low-carb diets and blood glucose control. There appears to be no health beneficial association in the total study population, and possibly diabetogenic effects in the Ghana study population. Pending verification and awaiting analysis on the quality of macronutrients (carbohydrates, total fats), low-carb diets cannot be promoted for the prevention of T2DM in Ghana. At the same time, our findings do not support the hypothesis that low-carb diets could accelerate the development of diabetes complications.

## Electronic supplementary material

Below is the link to the electronic supplementary material.

Supplementary Material 1
