# Supplementary material for: Associations of low-carb diets with glycaemic control and diabetic complications among adult Ghanaians: the RODAM study
Source: Eur J Nutr. 2025 Jun 7;64(5):207. doi: 10.1007/s00394-025-03729-3 (PMC12145308; doi:10.1007/s00394-025-03729-3)
Supplement: Supplementary file 1 — Supplementary Material 1 [file 394_2025_3729_MOESM1_ESM.docx]

**Figure S1. Flow chart for missing and imputed variables.**

Missing or implausible values for:

25 years < age > 70 years = 227

| Body Mass Index = 15 |
| --- |
| Waist circumference = 16 |
| LDL-cholesterol = 167 |
| HDL-cholesterol = 166 |
| Cholesterol = 163 |
| Triglycerides = 163 |
| Education = 385   \| Missing Ghana-FPQ data = 1192 \| \| --- \| \| Physical activity = 886 \| \| Smoking status = 406   \| Albuminuria = 0 \| \| --- \| \| Ankle-brachial index = 55 \| \| HbA1c = 406 \| \| Coronary artery disease = 464 \| \| Self-reported stroke = 461 \| \| Nephropathy = 184 \| \| |

No diabetes (n =5,236)

No type 2 diabetes

## Diabetes status

Participants (n = 6,385)

Diabetes (n = 662)

Self-reported diabetes and/or fasting plasma glucose >7.0 mmol/L and/or documented anti-diabetes medication or HbA1c ≥6.5% (≥53 mmol/mol)

## Complete-case analysis (n = 3,361)

Missing or implausible values for:

25 years < age > 70 years = 12

| Body Mass Index = 2 |
| --- |
| Waist circumference=4 |
| LDL-cholesterol = 30 |
| HDL-cholesterol = 30 |
| Cholesterol = 30 |
| Triglycerides = 30 |
| Education = 38 |
| Missing Ghana-FPQ data = 163 |
| Physical activity = 114 |
| Smoking status = 37   \| Albuminuria =0 \| \| --- \| \| Ankle-brachial index =10 \| \| HbA1c = 40 \| \| Coronary artery disease = 45   \| Self-reported stroke = 43 \| \| --- \| \| Nephropathy = 32 \| \| |

## Multiple imputation (n = 5,898)

Analysed (n = 2983)

Analysed (n = 378)

Did not attend physical examinations and/or blood sample collection (n = 487)

Included (n = 5,898)

**Figure S2.** Crude proportions (%) of self-reported microvascular and macrovascular complications among participants with type 2 diabetes, complete-case dataset (n=378). Error bars indicate 95% confidence intervals (CIs).

CAD: Coronary Artery Disease: PAD: Peripheral Artery Disease.

**Table S1. Characteristics of the RODAM study population by sex and study site, complete-case dataset (N = 3,361)**

| Characteristics | Total  (n=3,361) | Men  (n=1,250) | Women  (n=2,111) | Rural Ghana  (n=647) | Urban Ghana  (n=1315) | Amsterdam  (n=653) | Berlin  (n=436) | London  (n=310) |
| --- | --- | --- | --- | --- | --- | --- | --- | --- |
| Sex (female, %) | 62.8 | - | - | 61.7 | 71.9 | 59.4 | 45.0 | 59.0 |
| Age (years) | 46.0 ± 11.0 | 47.0 ± 11.3 | 45.5 ± 10.8 | 46.7 ± 12.7 | 45.2 ± 11.4 | 46.7 ± 8.5 | 45.2 ± 10.4 | 48.0 ± 10.8 |
| Education (%) | |  |  |  |  |  |  |  |
| Never or elementary | 37.3 | 20.8 | 47.0 | 57.4 | 43.7 | 36 | 9.4 | 9.0 |
| Low | 38 | 41.8 | 35.7 | 31.4 | 38.9 | 37 | 50.2 | 32.9 |
| Intermediate | 16.3 | 23.1 | 12.3 | 7.6 | 12.6 | 21.9 | 26.6 | 24.5 |
| Higher vocational | 8.5 | 14.4 | 5 | 3.8 | 4.8 | 5.2 | 13.8 | 33.6 |
| Body mass index (kg/m^2)^ | 26.8 ± 5.4 | 25.0 ± 4.4 | 27.9 ± 5.7 | 22.7 ± 4.4 | 26.9 ± 5.3 | 28.9 ± 4.9 | 27.6 ± 4.8 | 29.4 ± 4.9 |
| Waist circumference (cm) | 89.8 ± 12.5 | 87.2 ± 12.1 | 91.2 ± 12.5 | 81.1 ± 10.9 | 89.4 ± 11.6 | 94.8 ± 11.5 | 92.3 ± 11.6 | 95.2 ± 11.4 |
| T2DM (% positive) | 11.3 | 13.8 | 9.8 | 5.4 | 10.7 | 14.2 | 15.1 | 14.4 |
| HbA1c (mmol/mol) | 36.3 (31.5, 41.0) | 36.8 (31.3, 41.5) | 35.9 (31.5, 40.6) | 30.5 (26.3, 34.7) | 35.7 (31.6, 39.9) | 39.1 (35.7, 43.1) | 38.3 (34.6, 42.1) | 39.9 (35.7, 43.4) |
| Smoking (current or former, %) | 9.4 | 19.8 | 3.3 | 8.0 | 6.8 | 11.4 | 18.8 | 5.8 |
| Physical activity levels (MET-min/day) | 42  (6, 122) | 52  (10, 129) | 32  (4, 110) | 30  (3, 106) | 58  (21, 122) | 64  (20, 169) | 37  (3, 128) | 15  (5, 72) |
| Total Energy intake (kcal/day) | 2513 ± 827 | 2608 ± 848 | 2458 ± 808 | 2572 ± 823 | 2305 ± 662 | 2437 ± 840 | 2910 ± 931 | 2886 ± 936 |
| Carbohydrate intake (energy%) | 53.1 ± 9.0 | 52.3 ± 9.4 | 53.5 ± 8.7 | 55.9 ± 8.2 | 54.5 ± 8.1 | 51.2 ± 8.3 | 49.1 ± 10.8 | 50.9 ± 9.5 |
| Fat intake (energy%) | 32.4 ± 8.2 | 32.2 ± 8.7 | 32.4 ± 7.9 | 31.8 ± 7.3 | 31.6 ± 7.2 | 32.4 ± 8.3 | 34.1 ± 10.6 | 34.4 ± 9.5 |
| Protein intake (energy%) | 13.5 ± 2.6 | 13.5 ± 2.6 | 13.5 ± 2.6 | 11.6 ± 2.2 | 13.6 ± 2.4 | 14.9 ± 2.4 | 13.8 ± 2.5 | 14.1 ± 2.3 |
| Alcohol intake (g/day) | 0.1 (0.0, 1.9) | 0.8 (0.0, 5.4) | 0.1 (0.0, 1.1) | 0.1 (0.0, 1.2) | 0.1 (0.0, 0.06) | 1.4 (0.1, 0.04) | 2.0 (0.0, 11) | 0.1 (0.0, 1.8) |
| Serum total cholesterol (mmol/L) | 5.0 ± 1.1 | 4.9 ± 1.1 | 5.1 ± 1.1 | 4.6 ± 1.1 | 5.2 ± 1.2 | 5.0 ± 1.0 | 5.1 ± 1.0 | 5.1 ± 1.0 |
| Serum triglyceride (mmol/L) | 0.9 (0.7, 1.2) | 1.0 (0.7, 1.3) | 0.9 (0.7, 1.2) | 1.0 (0.7, 1.3) | 1.0 (0.7, 1.4) | 0.8 (0.6, 1.0) | 0.9 (0.6, 1.1) | 0.8 (0.6, 1.1) |
| Serum LDL-cholesterol (mmol/L) | 3.2 ± 1.0 | 3.2 ± 1.0 | 3.3 ± 1.0 | 2.8 ± 0.9 | 3.4 ± 1.0 | 3.2 ± 0.9 | 3.2 ± 0.9 | 3.3 ± 0.9 |
| Serum HDL-cholesterol (mmol/L) | 1.3 ± 0.4 | 1.3 ± 0.3 | 1.3 ± 0.4 | 1.2 ± 0.4 | 1.3 ± 0.3 | 1.4 ± 0.3 | 1.5 ± 0.3 | 1.4 ± 0.3 |
| Albuminuria category (%) |  |  |  |  |  |  |  |  |
| Normal:  <3 mg/mmol | 90.4 | 91.2 | 90.2 | 92.9 | 89.1 | 92.4 | 89.5 | 90.1 |
| Moderate:  3–30 mg/mmol | 8.2 | 7.2 | 8.7 | 6.3 | 9.3 | 6.9 | 9.4 | 8.6 |
| Severe:  ˃30 mg/mmol | 1.2 | 1.5 | 1.1 | 0.9 | 1.6 | 0.8 | 1.1 | 1.3 |
| CKD-EPI eGFR (mL/min/1.73 m^2^) | 94.8 ± 19.9 | 93.6 ± 19.1 | 95.6 ± 20.4 | 96.8 ±21.3 | 93.5 ± 19.8 | 95.1 ± 19.5 | 98.1± 18.9 | 91.4 ± 18.8 |

Continuous variables are expressed as means ± standard deviations (SDs) or medians (interquartile range, IQR). Categorical variables are expressed as numbers (percentages). CKD-eGFR: chronic kidney diseases according to estimated glomerular filtration rate; HDL: high-density lipoprotein; LDL: low-density lipoprotein; MET: metabolic equivalent of task; Type 2 diabetes was defined as fasting plasma glucose ≥7.0 mmol/L or HbA1c ≥48 mmol/mol (≥6.5%) or documented use of glucose-lowering medication or self-reported diabetes mellitus; HbA1c: glycated haemoglobin

**Table S2. Scoring criteria for determining the low-carb diet score, based on the distributions of macronutrients among individuals without type 2 diabetes in the complete-case dataset (n = 2,983).**

| **Score points** | **Carbohydrate intake (energy%)** | **Protein intake (energy%)** | **Total fat intake (energy%)** |
| --- | --- | --- | --- |
| 0 | >65.46 | <9.95 | <22.03 |
| 1 | 61.48-65.46 | 9.95-11.01 | 22.03-25.01 |
| 2 | 58.69-61.48 | 11.01-11.78 | 25.01-27.18 |
| 3 | 56.41-58.69 | 11.78-12.38 | 27.18-29.0 |
| 4 | 54.33-56.41 | 13.38-12.96 | 29.0-30.55 |
| 5 | 52.34-54.33 | 12.96-13.55 | 30.55-32.4 |
| 6 | 50.21-52.34 | 13.55-14.19 | 32.40-34.4 |
| 7 | 47.87-50.21 | 14.19-14.92 | 34.40-36.8 |
| 8 | 44.92-47.87 | 14.92-15.81 | 36.80-40.12 |
| 9 | 40.70-44.92 | 15.81-17.02 | 40.12-44.50 |
| 10 | <40.7 | >17.02 | >44.5 |

**Table S3. Pearson correlations between HbA1c, macronutrients, total energy, and the low-carb diet score**

|  | HbA1c (mmol/mol) | | Carbohydrate (energy%) | | Protein (energy%) | | Total fat (energy%) | | Total energy (kcal/d) | | Low-carb diet score | |
| --- | --- | --- | --- | --- | --- | --- | --- | --- | --- | --- | --- | --- |
|  | r | partial r | r | partial r | r | partial r | r | partial r | r | partial r | r | partial r |
| HbA1c (mmol/mol) | 1.000 | 1.000 |  |  |  |  |  |  |  |  |  |  |
| Carbohydrate (energy%) | 0.086 | 0.087 | 1.000 | 1.000 |  |  |  |  |  |  |  |  |
| Protein intake (energy%) | 0.169 | 0.170 | -0.369 | -0.313 | 1.000 | 1.000 |  |  |  |  |  |  |
| Fat intake (energy%) | 0.028 | 0.017 | -0.900 | -0.313 | 0.117 | 0.101 | 1.000 | 1.000 |  |  |  |  |
| Total energy (kcal/d) | -0.046 | -0.046 | 0.081 | -0.106 | -0.136 | -0.046 | -0.28 | 0.107 | 1.000 | 1.000 |  |  |
| Low-carb diet score | 0.107 | 0.059 | -0.921 | -0.917 | 0.626 | 0.589 | 0.809 | 0.822 | -0.007 | -0.041 | 1.000 | 1.000 |

**Table S4. Linear associations of low-carb diet score, total energy intake and the intakes of macronutrients with ln(HbA1c) among 3,361 Ghanaian adults, by diabetes status.**

| **ln(HbA1c)** | **SD** | **Crude model** | | **Model 1** | | **Model 2** | |
| --- | --- | --- | --- | --- | --- | --- | --- |
|  |  | **Std. ß (95% CI)** | **p-value** | **Std. ß (95% CI)** | **p-value** | **Std. ß (95% CI)** | **p-value** |
| **Diabetes (n=378)** | | |  |  |  |  |  |
| Low-carb diet score | 7.3 | -0.001 (-0.03, 0.03) | 0.976 | 0.02 (-0.02, 0.05) | 0.363 | 0.03 (-0.01, 0.07) | 0.184 |
| Total Energy **(**kcal/day) | 837 | -0.03 (-0.07, 0.01) | 0.114 | -0.02 (-0.06, 0.02) | 0.271 | 0.03 (-0.03, 0.10) | 0.327 |
| Carbohydrate (energy%) | 9.9 | 0.01 (-0.02, 0.04) | 0.678 | -0.01 (-0.04, 0.02) | 0.621 | -0.02 (-0.06, 0.02) | 0.311 |
| Protein (energy%) | 9.1 | 0.01 (-0.02, 0.04) | 0.479 | 0.03 (0.001, 0.07) | 0.087 | 0.03 (-0.01, 0.07) | 0.197 |
| Fat (energy%) | 2.6 | -0.01 (-0.04, 0.02) | 0.704 | -0.003 (-0.03, 0.03) | 0.986 | 0.02 (-0.02, 0.05) | 0.424 |
| **No diabetes (n=2,983)** | | |  |  |  |  |  |
| Low-carb diet score | 14.1 | 0.03 (0.02, 0.04) | <0.0001 | 0.02 (0.01, 0.02) | <0.0001 | 0.01 (0.01, 0.02) | <0.0001 |
| Total Energy (kcal/day) | 824 | 0.001 (-005.0, 0.006) | 0.838 | -0.001 (-0.01, 0.01) | 0.974 | 0.02 (0.004, 0.03) | 0.007 |
| Carbohydrate (energy%) | 8.9 | -0.02 (-0.3, -0.2) | <0.0001 | -0.01 (-0.01, 0.004) | 0.003 | -0.01 (-0.02, -0.003) | 0.004 |
| Protein (energy%) | 2.6 | 0.04 (0.03, 0.04) | <0.0001 | 0.02 (0.01, 0.02) | <0.0001 | 0.02 (0.01, 0.02) | <0.0001 |
| Fat (energy%) | 8.1 | 0.01 (0.01, 0.02) | <0.0001 | 0.01 (0.003, 0.01) | 0.0009 | 0.01 (0.003, 0.02) | 0.004 |

Standardized beta-coefficients (Std. β), their 95% confidence intervals (CIs), and p-values were calculated by linear regression. SD: standard deviation.

Model 1: adjusted for age (years), sex, and study site (5 categories).

Model 2: Model 1 + education (4 categories), smoking (yes/no), physical activity (METs-h/week), and dietary fibre (g/d).

**Table S5. Sensitivity analysis excluding study participants with self-reported diabetes mellitus. Linear associations of** **the low-carb diet score, total energy intake, and the intakes of macronutrients with ln (HbA1c) among 440 Ghanaian adults with screen-detected T2DM (imputed data set)**

| **ln(HbA1c)** |  | **Crude model** | | **Model 1** | | **Model 2** | |
| --- | --- | --- | --- | --- | --- | --- | --- |
|  |  | **ß (95% CI)** | **p-value** | **ß (95% CI)** | **p-value** | **ß (95% CI)** | **p-value** |
| **Diabetes (n=440)** | | |  |  |  |  |  |
| Low-carb diet score (per 1 SD) |  | -0.02 (-0.05, 0.003) | 0.081 | -0.004 (-0.03, 0.022) | 0.741 | -0.002 (-0.02, 0.03) | 0.929 |
| Energy intake (per 1 SD) (kcal/day) |  | -0.02 (-0.05,0.01) | 0.157 | -0.12 (-0.04,0.02) | 0.402 | 0.02 (-0.05,0.08) | 0.611 |
| Carbohydrate (per 1 SD) (energy%) |  | -0.03 (-0.05, 0.02) | 0.064 | -0.004 (-0.03, 0.02) | 0.775 | -0.001 (-0.03, 0.03) | 0.959 |
| Protein (per 1 SD) (energy%) |  | 0.002 (-0.03, 0.03) | 0.896 | 0.003 (-0.02, 0.03) | 0.819 | 0.03 (-0.003, 0.05) | 0.072 |
| Total fat (per 1 SD) (energy%) |  | -0.02 (-0.05, 0.01) | 0.180 | -0.01 (-0.03, 0.19) | 0.591 | -0.003 (-0.03, 0.03) | 0.855 |

Standardized beta-coefficients (Std. β), their 95% confidence intervals (CIs), and p-values were calculated by linear regression. SD: standard deviation.

Model 1: adjusted for age (years), sex, and study site (5 categories).

Model 2: Model 1 + education (4 categories), smoking (yes/no), physical activity (METs-h/week), and dietary fibre (g/d).

**Table S6. Linear associations of the low-carb diet score, total energy intake and the intakes of macronutrients with ln(HbA1c) among 3,361 Ghanaian adults, by study site**

| **ln(HbA1c)** | **SD** | **Ghana (n = 1,962)** | | **Europe (n = 1,399)** | | **p for interaction** |
| --- | --- | --- | --- | --- | --- | --- |
|  |  | **Std. ß (95% CI)** | **p-value** | **Std. ß (95% CI)** | **p-value** |  |
| **Diabetes (n=378)** | | **n = 174** |  | **n = 204** |  |  |
| Low-carb diet score | 7.3 | 0.06 (-0.003, 0.13) | 0.063 | -0.02 (-0.07, 0.03) | 0.509 | 0.011 |
| Total Energy (kcal/day) | 837 | -0.02 (-0.17, 0.14) | 0.833 | 0.04 (-0.02, 0.10) | 0.230 | 0.720 |
| Carbohydrate (energy%) | 9.9 | -0.07 (-0.14, -0.001) | 0.047 | 0.01 (-0.04, 0.05) | 0.717 | 0.031 |
| Protein (energy%) | 9.1 | 0.06 (-0.01, 0.13) | 0.077 | -0.01 (-0.06, 0.03) | 0.612 | 0.002 |
| Fat (energy%) | 2.6 | 0.06 (-0.01, 0.13) | 0.083 | -0.01 (-0.05, 0.03) | 0.583 | 0.193 |
| **No diabetes (n=2,983)** |  | **n = 1,788** |  | **n = 1,195** |  |  |
| Low-carb diet score | 14.1 | 0.01 (0.005, 0.02) | 0.003 | 0.001 (-0.01, 0.01) | 0.869 | <.0001 |
| Total Energy (kcal/day) | 824 | 0.01 (-0.01, 0.02) | 0.570 | -0.01 (-0.02, 0.01) | 0.301 | 0.874 |
| Carbohydrate (energy%) | 8.9 | -0.01 (-0.02, -0.004) | 0.004 | 0.003 (-0.01, 0.01) | 0.491 | <.0001 |
| Protein (energy%) | 2.6 | 0.01 (-0.001, 0.02) | 0.083 | 0.01 (0.003, 0.02) | 0.008 | <.0001 |
| Fat (energy%) | 8.1 | 0.02 (0.01, 0.02) | 0.002 | -0.003 (-0.01, 0.01) | 0.495 | 0.003 |

Standardized beta-coefficients (Std. β), their 95% confidence intervals (CIs), and p-values were calculated by linear regression. SD: standard deviation. The model was adjusted for age (years), sex, education (4 categories), smoking (yes/no), physical activity (METs-h/week), and dietary fibre (g/d).

**Table S7. Multiple-adjusted associations of the low-carb diet score with diabetes complications among participants with diabetes in the complete-case dataset (n=378)**

| **Outcome** | **Cases/**  **Controls** | **Crude** | | | | **Model 1** | | | | **Model 2** | | | |
| --- | --- | --- | --- | --- | --- | --- | --- | --- | --- | --- | --- | --- | --- |
|  |  | OR | (95% CI) | | p-value | OR | (95% CI) | | p-value | OR | (95%CI) | | p-value |
| Peripheral artery disease | 36/342 | 0.97 | (0.93, | 1.02) | 0.233 | 1.00 | (0.95, | 1.05) | 0.991 | 1.01 | (0.96, | 1.06) | 0.786 |
| Coronary artery disease | 55/323 | 0.98 | (0.94, | 1.02) | 0.269 | 1.00 | (0.96, | 1.04) | 0.834 | 1.00 | (0.96, | 1.05) | 0.938 |
| Self-reported stroke | 58/320 | 0.90 | (0.87, | 0.94) | <0001 | 0.93 | (0.89, | 0.98) | 0.003 | 0.93 | (0.87, | 0.98) | 0.004 |
| Nephropathy | 92/286 | 0.99 | (0.94, | 1.00) | 0.060 | 0.99 | (0.96, | 1.03) | 0.579 | 0.99 | (0.96, | 1.03) | 0.631 |

Odds ratios (ORs), their 95% confidence intervals (CIs), and p-values were calculated by logistic regression.

Model 1: adjusted for age (years), sex, study site (categorical).

Model 2: Model 1 + education (4 categories), energy intake (kcal/d), smoking (yes/no), physical activity (METs-h/week), and dietary fibre (g/d).

**Table S8. Sensitivity analysis excluding study participants with self-reported diabetes. Multiple-adjusted associations of the low-carb diet score with diabetes complications among 440 participants with screen-detected diabetes (imputed dataset)**

| **Outcome** | **Cases/**  **Controls** | **Crude** | | | | **Model 1** | | | | **Model 2** | | | |
| --- | --- | --- | --- | --- | --- | --- | --- | --- | --- | --- | --- | --- | --- |
|  |  | OR | (95% CI) | | p-value | OR | (95% CI) | | p-value | OR | (95%CI) | | p-value |
| Peripheral artery disease | 42/398 | 1.01 | (0.96, | 1.06) | 0.625 | 0.99 | (0.94, | 1.04) | 0.710 | 0.98 | (0.93, | 1.03) | 0.427 |
| Coronary artery disease | 49/391 | 1.01 | (0.96, | 1.06) | 0.761 | 1.02 | (0.97, | 1.07) | 0.505 | 1.03 | (0.98, | 1.09) | 0.226 |
| Self-reported stroke | 41/399 | 0.95 | (0.90, | 1.006) | 0.082 | 0.94 | (0.89, | 0.99) | 0.029 | 0.91 | (0.87, | 0.96) | 0.002 |
| Nephropathy | 110/286 | 1.04 | (1.002, | 1.07) | 0.041 | 1.03 | (0.99, | 1.06) | 0.160 | 1.03 | (0.99, | 1.06) | 0.204 |

Odds ratios (ORs), their 95% confidence intervals (CIs), and p-values were calculated by logistic regression.

Model 1: adjusted for age (years), sex, study site (categorical).

Model 2: Model 1 + education (4 categories), energy intake (kcal/d), smoking (yes/no), physical activity (METs-h/week), and dietary fibre (g/d).
